# A quality analysis of clinical anaesthesia study protocols from the Chinese clinical trials registry according to the SPIRIT statement

**DOI:** 10.18632/oncotarget.24982

**Published:** 2018-05-15

**Authors:** Lei Yang, Shouming Chen, Di Yang, Jiajin Li, Taixiang Wu, Yunxia Zuo

**Affiliations:** ^1^ Department of Anaesthesiology, West China Hospital, Sichuan University, Chengdu, China; ^2^ Department of Anaesthesiology, Second Affiliated Hospital, Zhejiang University School of Medicine, Hangzhou, China; ^3^ Department of Anaesthesiology, Sichuan Provincial People’s Hospital, Chengdu, China; ^4^ Medical Insurance Office, West China Hospital, Sichuan University, Chengdu, China; ^5^ Chinese Clinical Trials Registry, Chengdu, China; ^6^ Department of Anaesthesiology, West China Hospital, Sichuan University, Chengdu, China

**Keywords:** clinical study, protocol, anaesthesia, SPIRIT, quality analysis

## Abstract

**Objective:**

To learn about the overall quality of clinical anaesthesia study protocols from the Chinese Clinical Trials Registry and to discuss the way to improve study protocol quality.

**Methods:**

We defined completeness of each sub-item in SPIRIT as N/A (not applicable) or with a score of 0, 1, or 2. For each protocol, we calculated the proportion of adequately reported items (score = 2 and N/A) and unreported items (score = 0). Protocol quality was determined according to the proportion of reported items, with values >50% indicating high quality. Protocol quality was determined according to the proportion of reported items. For each sub-item in SPIRIT, we calculated the adequately reported rate (percentage of all protocols with score 2 and NA on one sub-item) as well as the unreported rate (percentage of all protocols with score 0 on one sub-item).

**Results:**

Total 126 study protocols were available for assessment. Among these, 88.1% were assessed as being of low quality. By comparison, the percentage of low-quality protocols was 88.9% after the publication of the SPIRIT statement. Among the 51 SPIRIT sub-items, 18 sub-items had an unreported rate above 90% while 16 had a higher adequately reported rate than an unreported rate.

**Conclusions:**

The overall quality of clinical anaesthesia study protocols registered in the ChiCTR was poor. A mandatory protocol upload and self-check based on the SPIRIT statement during the trial registration process may improve protocol quality in the future.

## INTRODUCTION

The overall quality of clinical trial reporting is considered to be inadequate [1], with limitations identified in the study design, conduct, and reporting stages [2, 3]. These deficiencies may be avoided by increasing the completeness and transparency of study protocols [4–6]. Therefore, the Standard Protocol Items: Recommendations for Intervention Trials (SPIRIT) statement was developed in 2013 [7] as an evidence-based guidance for the publication of clinical study protocols. The statement checklist contains 33 items and 51 sub-items under 8 headings. In addition to providing information on how to complete a study protocol, the SPIRIT statement also provides recommendations on important aspects of study design, conduct, and reporting [8].

It is a requirement for clinical trials to be registered on the World Health Organization International Clinical Trials Registry Platform (WHO ICTRP) (http://www.who.int/ictrp) [9, 10]. Of which the Chinese Clinical Trials Registry (ChiCTR) (www.chictr.org.cn) is a primary registry for clinical trials conducted in China as well as in other countries. Registered anaesthesia studies in ChiCTR have increased significantly in recent years. Despite researchers were encouraged to upload their study protocols during registered process in ChiCTR, there were no quality control of these protocols. Anaesthesia studies focus on anaesthesia technique as well as perioperative monitoring methods, which ensure patients’ safe and comfort following a surgical or medical procedure. Quality assessment of randomized controlled trial was performed in endodontics [11], but there is no similar quality assessment in anaesthesiology.

Therefore, to learn about the overall quality of clinical anaesthesia study protocols and to discuss the way to improve study protocol quality, we identified clinical trials in clinical anaesthesia from the ChiCTR and analyzed the protocols based on the 51 sub-items of the SPIRIT checklist.

## RESULTS

**Figure 1:**
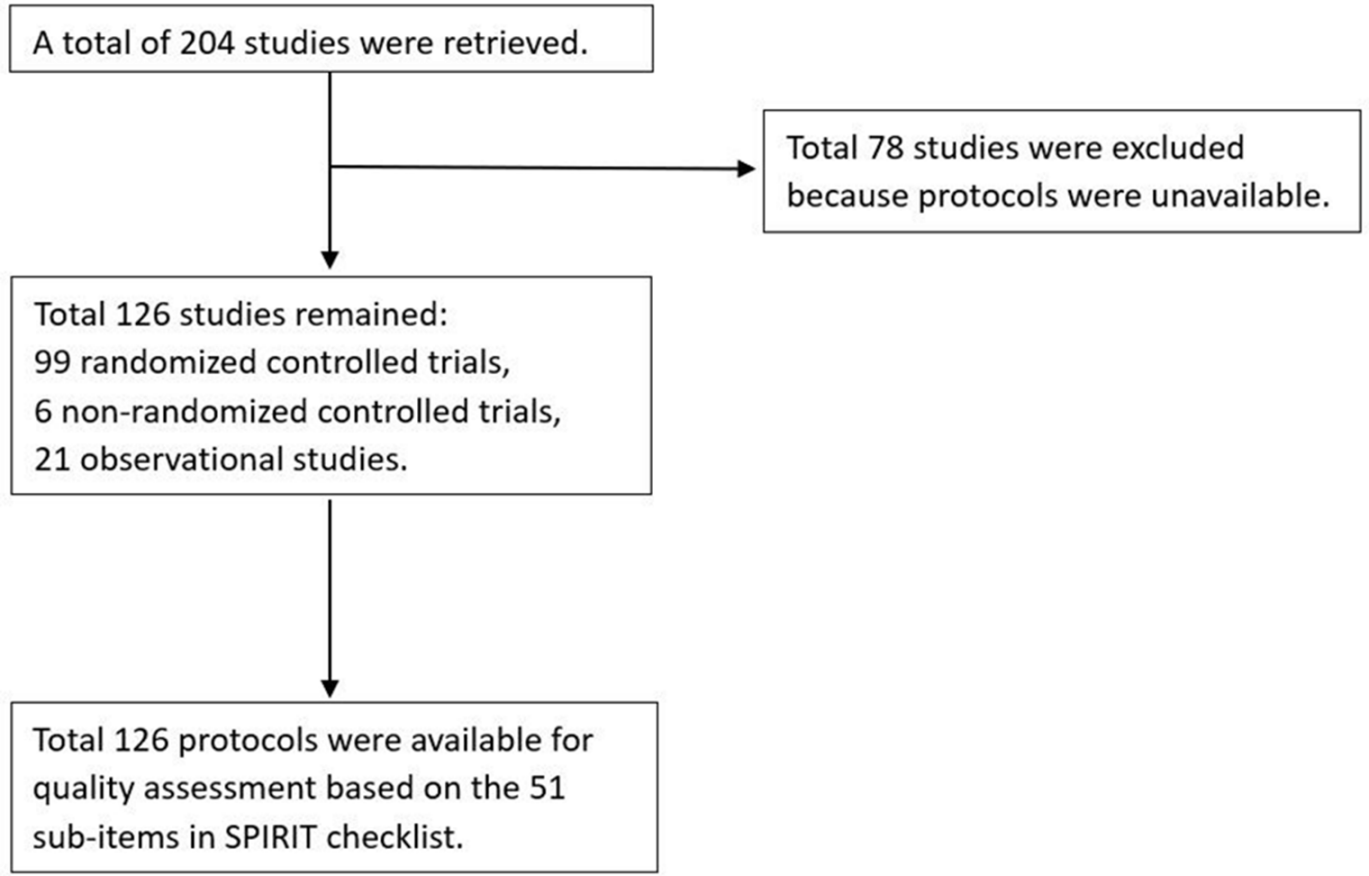
Flow chart

A total of 204 registered studies was retrieved from the ChiCTR, of which 126 study protocols were available for assessment. Among the 126 protocols there were 99 randomized controlled trials, 6 non-randomized controlled trials, and 21 observational studies (Figure 1).

### Overall quality assessment of protocols

**Figure 2:**
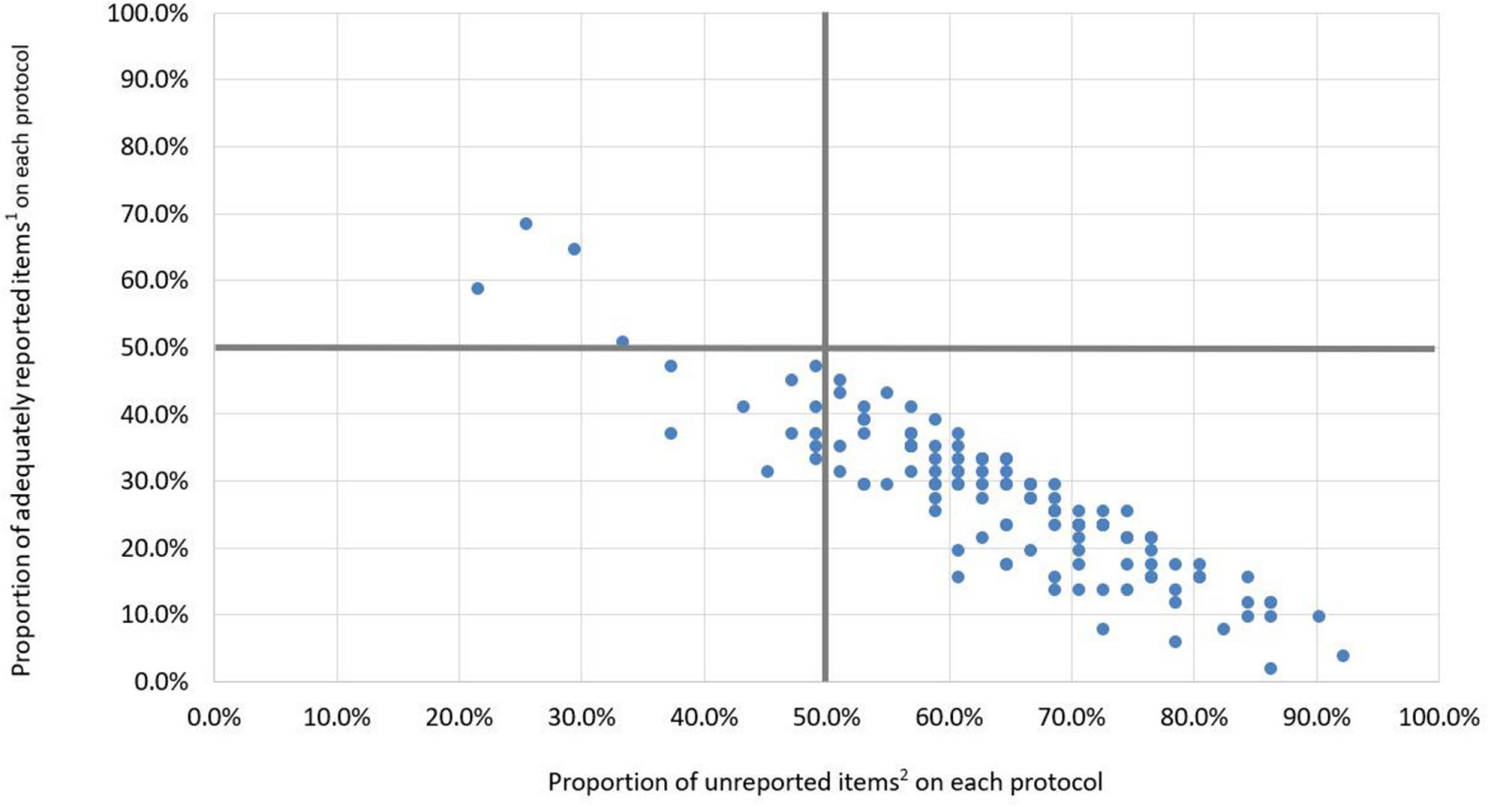
Distribution diagram of the quality of protocols (n=126) ^1^ Adequately reported items: sub-items assessed as having a score of 2 or N/A. ^2^ Unreported items: sub-items assessed as having a score of 0. Only 4 protocols (3.2%) were located in the upper left quadrant and were therefore of high quality (>50% of sub-items assessed as adequately reported), and 111 protocols (88.1%) were located in the lower right quadrant and were therefore of low quality (>50% of sub-items assessed as unreported), the remaining 11 protocols (8.7%) were located in the lower left quadrant and were of moderate quality.

Only 4 protocols (3.2%) were assessed as high quality and 111 protocols (88.1%) were assessed as low quality (Figure 2; Table 1). We identified only 18 protocols registered from the date of the SPIRIT statement to April 2015. Among these, 1 protocol (5.6%) was assessed as high quality, 1 (5.6%) was assessed as moderate quality, and 16 (88.9%) were low quality (Table 1).

**Table 1:** Quality of protocols before and after the SPIRIT statement was issued

	Number of high quality protocols	Number of moderate quality protocols	Number of low quality protocols	Total number of protocols
Before SPIRIT^#^ issued	3 (2.8%)	10 (9.2%)	95 (88.0%)	108
After SPIRIT^#^ issued	1 (5.6%)	1 (5.6%)	16 (88.9%)	18
Total	4 (3.2%)	11 (8.7%)	111 (88.1%)	126

^#^ SPIRIT: Standard Protocol Items: Recommendations for Intervention Trials.

### Analysis of SPIRIT sub-items

The following 18 sub-items had an unreported rate >90%: roles and responsibilities (5c, 5d), interventions (11c, 11d), data collection and methods (18b), data management (19), statistical methods (20c), data monitoring (21a, 21b), auditing (23), protocol amendments (25), declaration of interest (28), access to data (29), ancillary and post-trial care (30), dissemination policy (31a, 31b, 31c), and informed consent materials (32). A summary of the protocol assessment for each SPIRIT sub-item is shown in Table 2.

**Table 2:** Summary of protocol completeness assessment for each sub-item of the SPIRIT^#^ checklist (n=126)

	Item NO.	No. of protocols assessed as			
		Score 2^*^	Score 1^*^	Score 0^*^	N/A^*^
**Administrative information**					
Title	1	100 (79.4%)	20 (15.9%)	6 (4.8%)	0
Trial registration	2a	2 (1.6%)	23 (18.3%)	101 (80.2%)	0
	2b	1 (0.8%)	23 (18.3%)	102 (81.0%)	0
Protocol version	3	20 (15.9%)	16 (12.7%)	90 (71.4%)	0
Funding	4	13 (10.3%)	2 (1.6%)	111 (88.1%)	0
Roles and responsibilities	5a	29 (23.0%)	28 (22.2%)	69 (54.8%)	0
	5b	38 (30.2%)	19 (15.1%)	69 (54.8%)	0
	5c	2 (1.6%)	4 (3.2%)	120 (95.2%)	0
	5d	1 (0.8%)	2 (1.6%)	123 (97.6%)	0
**Introduction**					
Background and rationale	6a	96 (76.2%)	16 (12.7%)	14 (11.1%)	0
	6b	57 (45.2%)	36 (28.6%)	31 (24.6%)	2 (1.6%)
Objectives	7	111 (88.1%)	7 (5.6%)	8 (6.3%)	0
Trial design	8	76 (60.3%)	23 (18.3%)	26 (20.6%)	0
**Methods: Participants, interventions, and outcomes**					
Study setting	9	83 (65.9%)	4 (3.2%)	39 (31.0%)	0
Eligibility criteria	10	112 (82.4%)	8 (6.3%)	6 (4.8%)	0
Interventions	11a	105 (83.3%)	11 (8.7%)	10 (7.9%)	0
	11b	29(23.0%)	9 (7.1%)	88 (69.8%)	0
	11c	6 (4.8%)	3 (2.4%)	117 (92.9%)	0
	11d	5 (4.0%)	5 (4.0%)	116 (92.1%)	0
Outcomes	12	111 (88.1%)	12 (9.5%)	3 (2.4%)	0
Participant timeline	13	34 (27.0%)	1 (0.8%)	91 (72.2%)	0
Sample size	14	28 (22.2%)	60 (47.6%)	38 (30.2%)	0
Recruitment	15	15 (11.9%)	29 (23.0%)	82 (65.1%)	0
**Methods: Assignment of interventions (for controlled trials)**					
Allocation:					
Sequence generation	16a	30 (23.8%)	21 (16.7%)	69 (54.8%)	6(4.8%)
Allocation concealment mechanism	16b	11 (8.7%)	15 (11.9%)	92 (73.0%)	8(6.3%)
Implementation	16c	4 (3.2%)	12 (9.5%)	103 (81.7%)	7(5.6%)
Blinding	17a	20 (15.9%)	8(6.3%)	33 (26.2%)	65 (51.6%)
	17b	6 (4.8%)	1 (0.8%)	54 (42.9%)	65 (51.6%)
**Methods: Data collection, management and analysis**					
Data collection methods	18a	11 (8.7%)	31 (24.6%)	84 (66.7%)	0
	18b	3 (2.4%)	7(5.6%)	116 (92.1%)	0
Data management	19	8 (6.3%)	4 (3.2%)	114 (90.5%)	0
Statistical methods	20a	59 (46.8%)	13 (10.3%)	54 (42.9%)	0
	20b	6(4.8%)	8 (6.3%)	112 (88.9%)	0
	20c	3 (2.4%)	4 (3.2%)	119 (94.4%)	0
**Methods: Monitoring**					
Data monitoring	21a	6(4.8%)	2 (1.6%)	118 (93.7%)	0
	21b	3 (2.4%)	1 (0.8%)	122 (96.8%)	0
Harms	22	33 (26.2%)	10 (7.9%)	83 (65.9%)	0
Auditing	23	1 (0.8%)	1 (0.8%)	124 (98.4%)	0
**Ethics and dissemination**					
Research ethics approval	24	61 (48.4%)	10 (7.9%)	55 (43.7%)	0
Protocol amendments	25	10 (7.9%)	2 (1.6%)	114 (90.5%)	0
Consent or assent	26a	78 (61.9%)	9 (7.1%)	39 (31.0%)	0
	26b	1 (0.8%)	2 (1.6%)	51 (40.5%)	72 (57.1%)
Confidentiality	27	14 (11.1%)	3 (2.4%)	108 (85.7%)	0
Declaration of interests	28	1 (0.8%)	3 (2.4%)	122 (96.8%)	0
Access to data	29	7 (5.6%)	5 (4.0%)	114 (90.5%)	0
Ancillary and post-trial care	30	4 (3.2%)	4 (3.2%)	118 (93.7%)	0
Dissemination policy	31a	4 (3.2%)	3 (2.4%)	119 (94.4%)	0
	31b	0	2 (1.6%)	124 (98.4%)	0
	31c	0	1 (0.8%)	125 (99.2%)	0
**Appendices**					
Informed consent materials	32	3 (2.4%)	0	123 (97.6%)	0
Biological specimen	33	1 (0.8%)	0	49 (38.9%)	76(60.3%)

^#^ SPIRIT: Standard Protocol Items: Recommendations for Intervention Trials. ^*^A score of 2 indicated that all important information was adequately reported in the protocol for that sub-item; a score of 1 indicated that some important information was inadequately reported; a score of 0 indicated that no information related to the sub-item was reported. N/A indicated that the item was not applicable to the specific protocol.

**Figure 3:**
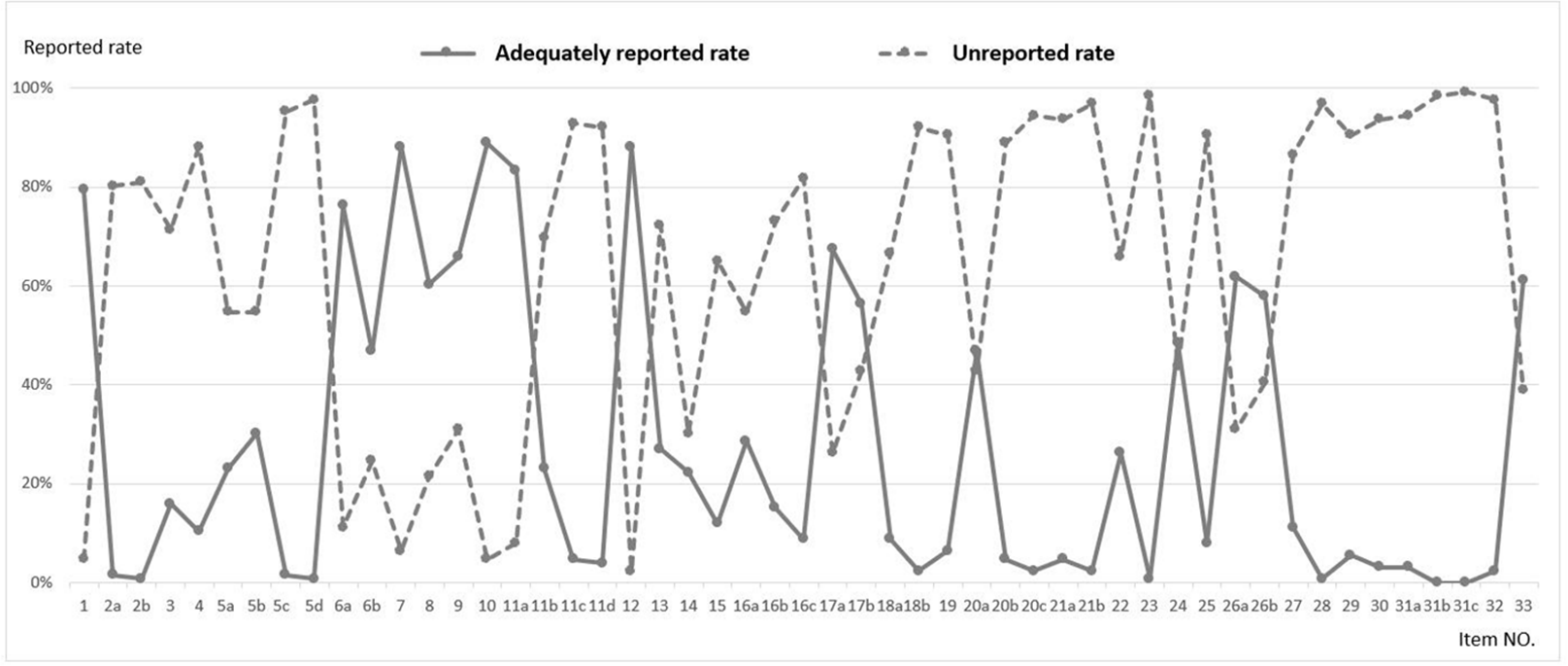
Adequately reported rate^1^ and unreported rate^2^ for each SPIRIT^#^ sub-item (n=126) ^1^ Adequately reported rate was the percentage of protocols with a score of 2 or NA for one sub-item among all protocols assessed. ^2^ Unreported rate was the percentage of protocols with a score of 0 for one sub-item among all protocols assessed. # SPIRIT: Standard Protocol Items: Recommendations for Intervention Trials. The adequately reported rate was lower than the unreported rate for 35 of the 51 sub-items.

The following 16 sub-items had an adequately reported rate greater than the unreported rate: title (1), background and rationale (6a, 6b), objectives (7), trial design (8), study setting (9), eligibility criteria (10), intervention (11a), outcomes (12), blinding (17a, 17b), statistical methods (20a), research ethics approval (24), consent or assent (26a, 26b), and biological specimen (33) (Figure 3).

## DISCUSSION

The completeness of protocol was one of the factors which determined clinical study quality. Protocol upload was required during registered process in ChiCTR, but not in other registry. So the anaesthesia study protocols in ChiCTR had a certain representation. Unfortunately, the overall quality of most anaesthesia study protocols submitted to the ChiCTR was poor.

To improve study quality, it has been a requirement of most clinical journals that clinical trials are registered in international databases, although current trial registry requirements appear to be insufficient [12]. Submission of a detailed protocol could help to prevent inadequate trial conduct and unnecessary protocol amendments [13]. However, this is not a mandatory requirement during the trial registration process and only 61.7% of anaesthesia study protocols in our review were available in the ChiCTR. This indicates a lack of awareness among some researchers regarding the importance of the study protocol. We suggest that a mandatory requirement to provide the protocol and perform a self-check of completeness according to the SPIRIT statement during the registration process may improve the quality of clinical studies.

The SPIRIT statement was issued at the end of 2013 [7, 14]. However, we did not observe a significant improvement in the quality of protocols registered after this date (Table 1). Information related to the sub-items in the Administrative information, Introduction, Ethics and dissemination, and Appendices sections should be reviewed and confirmed during the study planning stage. Missing information within these sections may thus be the result of a lack of awareness among researchers about the SPIRIT statement. Therefore, to increase protocol completeness and quality, information on the SPIRIT statement and other relevant programs such as the *Research to Publication* initiative [15] should be widely disseminated.

Regarding the detailed content of the SPIRIT statement in our study, 16 sub-items were found to have an adequately reported rate greater than the unreported rate, with the remaining 35 sub-items requiring further information (Figure 3). Among these 35 sub-items, both the Participant timeline and Recruitment fields were frequently overlooked. Insufficient recruitment is a common feature of clinical trials, [16–19] and can prolong the study period, increase research costs, or reduce the statistical power. Increased focus on participant timelines and recruitment strategies during the study planning stage has been shown to improve recruitment and minimize loss to follow-up [8]. However, in the current study, only 27.0% of protocols adequately reported the participant timeline and only 11.9% adequately reported recruitment. In addition, data collection, management, and monitoring have been identified as important determinants of study quality and participant safety [20, 21]. However, we found that these items were adequately reported in only 10% of protocols. Increased use of the SPIRIT guidelines will allow more researchers to recognize the benefits of establishing detailed protocols prior to study initiation. Meanwhile, adequate clinical research training on methodology, epidemiology, and statistics may improve the completeness of protocols and subsequently enhance the quality of clinical trials.

The limitation of our study is that only 61.8% of study protocol were available. Due to the low percentage, the result of our study may be biased. But even if we assumed that the rest unavailable protocols were high quality, the overall quality of anaesthesia protocols was still poor.

## MATERIALS AND METHODS

We searched the ChiCTR for studies registered from 2005 to April 2015 using the term ‘anaesthesia’ in the title or ‘anaesthesiology department’ in the affiliated institution name. Study protocols were obtained from ChiCTR, and two of the authors (DY and SC) independently assessed the completeness of protocols based on the 51 sub-items of the SPIRIT checklist [7]. Any disagreement was resolved by discussion with the third author (LY).

We defined three levels of completeness for each sub-item, scored as 2, 1, and 0. A score of 2 was given where all important information was adequately reported in the protocol for the sub-item. A score of 1 meant that some of the important information was inadequately reported. A score of 0 indicated that none of the important information was reported. N/A (not applicable) was used where the sub-item was not applicable to the protocol.

Quality analysis was performed in two steps:

1. Overall quality assessment of protocols

For each protocol, we calculated the proportion of adequately reported items (sub-items with a score of 2 or N/A) and unreported items (sub-items with a score of 0). Protocols were considered high quality if the proportion of adequately reported items was more than 50% or low quality if the proportion of unreported items was more than 50%; the remaining protocols were considered moderate quality.

2. Analysis of SPIRIT sub-items

We analyzed the reporting rate for each sub-item in the SPIRIT checklist by calculating the adequately reported rate (percentage of scores = 2 or NA for a sub-item across all protocols) as well as the unreported rate (percentage of scores = 0 for a sub-item across all protocols).

## CONCLUSIONS

The quality of anaesthesia study protocols in the ChiCTR was assessed as poor in the current study. This issue might be addressed by introducing a mandatory requirement to provide the study protocol during the registration process along with performing a self-check of the protocol according to the SPIRIT guidelines. Awareness of the SPIRIT statement and adequate clinical research training should also be ensured within the clinical anaesthesia research community.

## Abbreviations

SPIRIT: Standard Protocol Items: Recommendations for Intervention Trials
WHO ICTRP: World Health Organization International Clinical Trials Registry Platform
ChiCTR: Chinese Clinical Trials Registry
N/A: Not applicable
